# Supplementary material for: Obesity and Cancer Rehabilitation for Functional Recovery and Quality of Life in Breast Cancer Survivors: A Comprehensive Review
Source: Cancers (Basel). 2024 Jan 25;16(3):521. doi: 10.3390/cancers16030521 (PMC10854903; doi:10.3390/cancers16030521)
Supplement: Supplementary file 1 [file cancers-16-00521-s001.zip › cancers-2816488-supplementary.pdf]

Scale for the Assessment of Narrative Review Articles – SANRA

Please rate the quality of the narrative review article in question, using categories 0–2 on the following scale. For each aspect of quality, please choose the option which best fits your evaluation, using categories 0 and 2 freely to imply general low and high quality. These are not intended to imply the worst or best imaginable quality.

1) Justification of the article's importance for the readership

The importance is not justified.	0	2
The importance is alluded to, but not explicitly justified.	1	
The importance is explicitly justified.	2	

2) Statement of concrete aims or formulation of questions

No aims or questions are formulated.	0	2
Aims are formulated generally but not concretely or in terms of clear questions.	1	
One or more concrete aims or questions are formulated.	2	

3) Description of the literature search

The search strategy is not presented.	0	2
The literature search is described briefly.	1	
The literature search is described in detail, including search terms and inclusion criteria.	2	

4) Referencing

Key statements are not supported by references.	0	2
The referencing of key statements is inconsistent.	1	
Key statements are supported by references.	2	

5) Scientific reasoning

(e.g., incorporation of appropriate evidence, such as RCTs in clinical medicine)

The article's point is not based on appropriate arguments.	0	2
Appropriate evidence is introduced selectively.	1	
Appropriate evidence is generally present.	2	

6) Appropriate presentation of data

(e.g., absolute vs relative risk; effect sizes without confidence intervals)

Data are presented inadequately.	0	2
Data are often not presented in the most appropriate way.	1	
Relevant outcome data are generally presented appropriately.	2	

Sumscore 12
